# Assessment of atherosclerotic risk among patients with epilepsy on valproic acid, lamotrigine, and carbamazepine treatment

**DOI:** 10.17712/nsj.2017.2.20160342

**Published:** 2017-04

**Authors:** Nudrat A. Zuberi, Mukhtiar Baig, Shazia Bano, Zehra Batool, Saeeda Haider, Tahira Perveen

**Affiliations:** *From the Department of Biochemistry (Zuberi), Unaizah College of Medicine, Qassim, the Department of Clinical Biochemistry (Baig), Faculty of Medicine, King Abdulaziz University, Jeddah, Kingdom of Saudi Arabia, the Department of Biochemistry (Bano), Jinnah Postgraduate Medical Center, the Department of Biochemistry (Batool), Jinnah University for Women, and the Neurochemistry and Biochemical Neuropharmacology Research Unit (Haider, Perveen), Department of Biochemistry, University of Karachi, Karachi, Pakistan*

## Abstract

**Objective:**

To compare the long-term effects of carbamazepine (CBZ), valproic acid (VPA), and lamotrigine (LTG) as monotherapy on the markers of vascular risk.

**Methods:**

The present cross-sectional study was carried out at the Department of Neurology, Jinnah Postgraduate Medical Centre (JPMC), Karachi, Pakistan, from 2012 to 2013. We selected 120 adult patients with epilepsy and 40 control subjects. The patients with epilepsy were divided into 3 groups according to the use of antiepileptic drugs (AEDs) (CBZ, n = 40; VPA, n = 40; and LTG, n = 40). All participants’ total cholesterol (TC), triglycerides (TG), low-density lipoprotein cholesterol (LDL-c), very low-density lipoprotein cholesterol (VLDL-c), high-density lipoprotein cholesterol (HDL-c), ratio of TC/HDL-c, ratio of LDL-c/HDL-c, body mass index (BMI), and blood pressure was determined.

**Results:**

In patients with epilepsy, CBZ and VPA treatment caused a noteworthy increase in the concentrations of TG, TC, and LDL-c compared with LTG treatment and the control group (*p*<0.001). The HDL-c significantly decreased in CBZ, VPA, and LTG-treated patients as compared with controls (*p*<0.001). The ratio of LDL-c/HDL-c and TC/HDL-c significantly increased in VPA- and CBZ-treated groups compared with the LTG-treated, and control group, while the ratio was also considerably elevated in patients treated with CBZ as compared with the patients treated with VPA. The weight and BMI of the patients treated with AEDs were higher (*p*<0.01).

**Conclusion:**

Patients with epilepsy on CBZ or VPA have changed vascular risk markers that may lead to atherosclerosis, while LTG-treated patients have less alteration in lipid profile.

Epilepsy is a chronic non-communicable neurological disorder that influences people of all ages, races, and social class from all over the world. There are more than 50 million people in the world suffering from epilepsy; 80% of these people live in developing regions, and, annually, 2.4 million new cases arise.^1^ It is reported that with proper diagnoses and treatment three-fourths of patients could lead normal lives. It was estimated that in Pakistan the prevalence of epilepsy is approximately 10 per 1000 people.^2^ Epileptic subjects receive long-term treatment with AEDs; this may cause an alteration in serum lipids and lipoproteins and thus increase the risk of atherosclerosis in epileptic patients. Derangement in lipid profile is considered a significant threatening factor for atherosclerosis.^3^ Use of certain AEDs has been linked with an augmented risk of atherosclerosis, suggesting that there could be increased chances of ischemic heart disease in patients with epilepsy.^4^,^5^ A recent study concluded that the process of atherosclerosis increases in patients with epilepsy treated with AEDs, whether enzyme inducer or enzyme inhibitor AEDs.^6^ Derangement in lipid profile by valproic acid (VPA) treatment is debatable; it is reported that it may decrease, increase, or have no effect on lipid profile.^5^,^7^-^10^ In the literature, there is disagreement regarding the effect of AED therapy on the advancement of atherosclerosis. There is very limited literature on this topic in this part of the world, while most of the published studies have been conducted in Western countries. However, there is a difference in our diet, culture, and genetics from the Western world; hence, some studies on the consequences of AEDs treatment in our local population were essential. Therefore, this study was designed to compare the long-term effects of carbamazepine (CBZ), VPA, and lamotrigine (LTG) as monotherapy on the markers of vascular risk.

## Methods

The present cross-sectional study was carried out at the Department of Neurology, Jinnah Postgraduate Medical Centre (JPMC), Karachi, Pakistan, which is a tertiary care hospital, from 2012 to 2013. We selected 120 adult patients with epilepsy from the outpatient clinic by the convenience sampling technique (55 males and 65 females). The epileptic patients were divided into 3 groups according to the use of antiepileptic drug (AED) (CBZ, n = 40, 18 males and 22 females; VPA, n = 40, 18 males and 22 females; and LTG, n = 40, 19 males and 21 females). All included patients used AED as monotherapy for at least 2 years before the start of the study. The people who were on other AEDs, any other regular medication, alcoholics, and pregnant/lactating females were excluded. Patients with a history of ischemic heart diseases, hypertension, diabetes mellitus, cigarette smoking, endocrine disorders, or autoimmune diseases, or receiving medication that could affect blood lipid levels were also excluded. Physical examination of all studied subjects was performed, and age, duration, onset, etiology of epilepsy, and duration of AED were noted. Forty healthy, age- and gender-matched individuals (26 male and 14 female) were recruited from the general population in the control group. The Ethical committee of JPMC, Karachi, approved the present study, and written consent was taken from all patients and control subjects.

Five milliliters of blood was drawn from an antecubital vein from each patient, and after centrifugation the serum was analyzed for lipid profile. All samples were collected after 12-14 hours of fasting. The measurement of total cholesterol (TC), triglycerides (TG), and high-density lipoprotein cholesterol (HDL-c) was carried out using the Cholesterol Oxidase Phenol Aminophenazone method, and kits were supplied by Randox Laboratories private limited (London, England). Very low-density lipoprotein cholesterol (VLDL-c) and low-density lipoprotein cholesterol (LDL-c) were calculated using the Friedewald formula that is, LDL-c = TC - (HDL-c + TG/5) and VLDL-c = TG/5.

The data were analyzed using the Statistical Package for Social Sciences (SPSS Inc., Chicago, IL, USA) version 16. Comparison between the basic characteristics of the control group and patients with epilepsy in the 3 groups, and biochemical parameters among all 4 groups was carried out by one-way analysis of variance (ANOVA), and the categorical variable (gender) was compared by a Chi-Square test. A *p*-value <0.05 was taken as the significant level.

## Results

**Figure 1:**
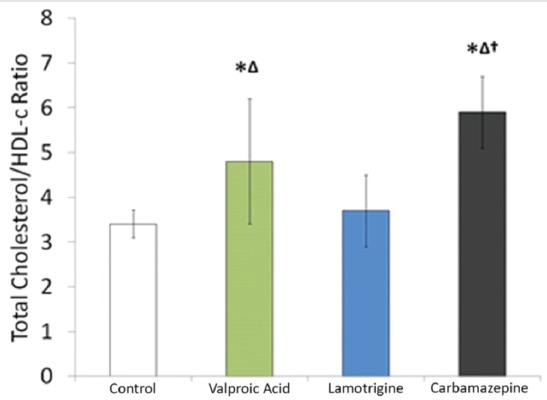
- Comparison of total cholesterol/high-density lipoprotein cholesterol ratio among the controls and valproic acid (VPA), lamotrigine (LTG), and carbamazepine treated patients. *Significant as compared with controls (*p*<0.05), ∆significant as compared with LTG (*p*<0.05), †significant as compared with VPA (*p*<0.05). The line on each bar indicates standard deviation.

**Figure 2:**
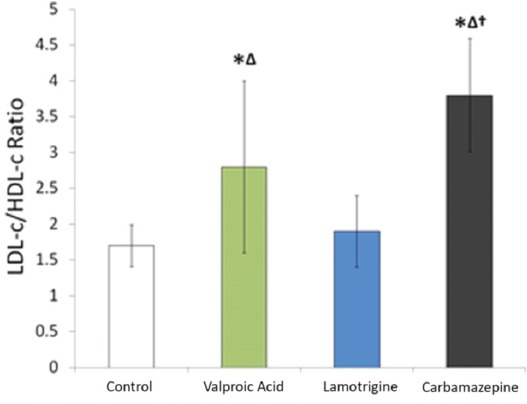
- Comparison of low-density lipoprotein cholesterol/high-density lipoprotein cholesterol ratio among the controls and valproic acid (VPA), lamotrigine (LTG), and carbamazepine treated patients. *Significant as compared with controls (*p*<0.05), ∆significant as compared with LTG (*p*<0.05), †significant as compared with VPA (*p*<0.05). The line on each bar indicates standard deviation.

The comparison of baseline characteristics of the patients in all 4 groups is shown in **Table 1**. There was a significant difference in the duration of treatment (*p*<0.01). The body mass index (BMI) and body weight of the antiepileptic treated groups was notably high (*p*<0.01) compared with the age-matched control group (**Table 1**). The concentrations of TG, TC, and LDL-c significantly increased in VPA- and CBZ-treated patients compared with LTG-treated and control groups (*p*<0.001), while no significant change was observed between LTG-treated and control groups. The HDL-c significantly decreased in VPA, CBZ, and LTG-treated patients compared with controls (*p*<0.001). The levels of VLDL-c considerably increased in CBZ-treated patients compared with the VPA and LTG-treated groups and controls (*p*<0.001), while, in the VPA-treated patients, it was higher than the LTG-treated group (*p*<0.001) (**Table 2**). The ratio of TC/HDL-c and LDL-c /HDL-c significantly increased in VPA- and CBZ-treated groups compared with controls and the LTG-treated group, and the ratio also significantly increased in the CBZ-treated group compared with the VPA-treated group (**Figures 1 & 2**).

**Table 1:** Comparison of basic characteristics of the controls and patients receiving valproic acid, carbamazepine, and lamotrigine treatment.

Parameters	Patients treated with different AEDs				*P*-value
	Valproic acid (n=40)	Carbamazepine (n=40)	Lamotrigine (n=40)	Controls (n=40)	
Duration of treatment	4.9±3.82	4.2±3.67	7.3±4.71	---	0.01*
Age	32.25±5.39	31.67±4.32	33.12±6.86	31.62±5.73	0.29
*Gender*					
Male	18	18	19	26	
Female	22	22	21	14	0.89^#^
BMI	24.71±5.36	25.21±4.10	26.27±6.18	21±2.63	<0.001*
Systolic blood pressure (mm Hg)	116.12±15.72	118.0±13.37	116.0±13.51	116.8±9.30	0.64
Diastolic blood pressure (mm Hg)	76.21±10.17	76.50±8.77	75.10±9.11	73.8±7.82	0.15

* Significant (*p*<0.05),

# Chi-Square test, values are shown as mean + SD (except gender), AEDs - antiepileptic drugs, BMI - body mass index

**Table 2:** Comparison of lipid profile among the control and valproic acid, carbamazepine, and lamotrigine treated patients.

Parameters	Patients treated with different AEDs				*P*-value
	Valproic acid (n=40)	Carbamazepine (n=40)	Lamotrigine(n=40)	Controls (n=40)	
Total cholesterol (mg/dl)	170.7±42.17^*∆^	212.7±31.89^*∆†^	123.1±18.72	141.5±13.31	0.001
Triglyceride (mg/dl)	153.5±43.94^*∆^	203.0±48.82^*∆†^	119.9±25.97	140.5±18.38	0.001
HDL-c (mg/dl)	36.2±3.62^*^	35.7±3.51^*^	33.0±4.26^*^	41.6±3.24	0.001
LDL-c (mg/dl)	101.6±37.2^*∆^	135.0±28.1^*∆†^	64.8±17.64	71.7±12.78	0.001
VLDL-c(mg/dl)	30.7±8.77^∆^	40.9±9.59^*∆†^	24.1±5.18	28.2±3.52	0.001

* Significant as compared with controls (*p*<0.05),

∆ significant as compared to lamotrigine (*p*<0.05),

† Significant as compared to VPA (*p*<0.05), AEDs - antiepileptic drugs, HDL-c - high-density lipoprotein cholesterol, LDL-c - low-density lipoprotein cholesterol,

VLDL-c - very low-density lipoprotein cholesterol

## Discussion

In the present study, epileptic patients had significantly increased body weight and BMI. Numerous studies have reported variable effects of AEDs on body weight. Three studies observed weight gain with VPA,^11^-^13^ and both weight gain^14^ and no change in weight^12^,^13^ have been linked with CBZ. However, no change in weight was reported with LTG.^13^,^15^ In our study, it was observed that the levels of TC, TG, and LDL-c significantly increased in CBZ- and VPA-treated patients compared with the LTG-treated and control groups, while no substantial difference was observed between LTG-treated and control groups. Several similar and dissimilar results were found in the literature. Results of several studies have shown that CBZ and phenytoin (PHT) (enzyme-inducing AEDs) significantly elevate the levels of TC, LDL-c, and triglycerides.^7^,^16^,^17^ They further suggested that enhancement in the thickness of intima-media of the common carotid artery in patients treated with PHT or CBZ could be due to deposition of TC and LDLs. Chuang et al^7^ reported that the length of treatment with CBZ, PHT, and VPA shows a significant difference in the speeding up of atherosclerosis, and constitutes the main role in this process.^7^

The alteration in lipid profile is considered a significant threatening factor for atherosclerosis because elevated levels of LDLs play an imperative role in atherosclerotic development by increasing endothelial permeability, withholding lipoproteins inside the intima of blood vessels, conscripting inflammatory cells, and creating foam cells.^3^,^18^ In the present study, the results of LTG effects on lipid profile are similar to other studies^7^,^19^,^20^ these studies also showed no effects of LTG on lipid profile. Moreover, a study reported an improvement in vascular risk factors when a patient is shifted from an enzyme-inducing AED to LTG (a non-enzyme inducing AED).^19^

A few studies documented no noteworthy differences in lipid profile parameters among the different AED groups; only HDL-c level reduced in patients taking VPA.^21^,^22^ In the epileptic literature, derangement in lipid profile by VPA treatment is found debatable.^5^,^7^-^10^ Some studies observed no effect,^7^,^8^ while others established a noteworthy alteration in lipid profile.^9^,^10^,^23^,^24^ One study^9^ reported a considerable elevation in total cholesterol and LDLs after 12 months of VPA treatment, but levels of TG and HDLs remained the same.^9^ In our study, the HDL-c significantly decreased in VPA-, CBZ- and LTG-treated patients compared with controls. The present results are compatible with several studies.^21^,^22^

The deranged lipid levels in adult patients with epilepsy can be explained by the fact that such patients usually do less exercise and are physically less active, and this could be a reason for low HDL-c concentration in epileptic patients. People with epilepsy, and sometimes physicians themselves are reluctant to include physical exercise programs as a supportive treatment because of the fear that exercise would trigger seizures, or it may be due to lack of information.^25^ On the contrary, the literature recommends inclusion of exercise while treating epileptic patients because it has a positive outcome for epileptic control and for improving the quality of life of epileptic subjects.

The ratio of TC/HDL-c and LDL-c/HDL-c significantly increased in the VPA- and CBZ-treated groups compared with controls and the LTG-treated group, while the ratio also significantly increased in the CBZ-treated group compared with the VPA-treated group. It is considered that the ratio amongst the different fractions of cholesterol (TC/HDL-c; LDL-c/HDL-c) is an important forecaster of atherosclerosis development. Decreased ratios of TC/HDL-c and LDL-C/HDL-c have potent protecting effects against atherosclerosis and vice versa.^26^ In our epileptic patients, the elevated ratios of TC/HDL-c and LDL-c/HDL-c indicate a higher atherosclerosis risk.

Diet affects the lipid profile levels; therefore, to exclude the influence of diet, we collected all samples from only one public sector hospital. In a country like Pakistan, for several reasons, almost all people who use the public sector hospitals’ facilities are from the low socio-economic class. Therefore, their dietary patterns are almost similar, and it seems that the change in lipid profile in patients with epilepsy was due to AEDs.

There are several limitations to our study. First, we did not measure other biochemical vascular risk factors. Second, the thickness of the intima-media of the carotid artery was not determined because of unavailability of the facilities. Third, a history of the subject’s physical activities could not be taken. Fourth, detailed information on possible differences in dietary habits among groups was not collected, and it is also a single center study so our results cannot be generalized.

In conclusion, patients with epilepsy on CBZ or VPA have changed vascular risk markers that may lead to atherosclerosis while LTG-treated patients have less alteration in lipid profile. Antiepileptic drug treatment also increases body weight and BMI. Hence, careful monitoring of body weight and lipid profile is needed to detect and manage any significant change. The choice of drug when treating patients should also be taken into consideration.
